# Geochemical evidence for the link between sulfate reduction, sulfide oxidation and phosphate accumulation in a Late Cretaceous upwelling system

**DOI:** 10.1186/s12932-015-0017-1

**Published:** 2015-04-10

**Authors:** Heiko Alsenz, Peter Illner, Sarit Ashckenazi-Polivoda, Aaron Meilijson, Sigal Abramovich, Shimon Feinstein, Ahuva Almogi-Labin, Zsolt Berner, Wilhelm Püttmann

**Affiliations:** Department of Environmental Analytical Chemistry, Goethe-University, Institute of Atmospheric and Environmental Sciences, Altenhoeferallee 1, 60438 Frankfurt am Main, Germany; Institute for Mineralogy and Geochemistry, Karlsruhe University, 76131 Karlsruhe, Germany; Dead Sea and Arava Science Center, Neve Zohar, Dead Sea Mobile Post, Neve Zohar, 86910 Israel; Department of Geological and Environmental Sciences, Ben-Gurion University of the Negev, POB 653, Beer Sheva, 84105 Israel; Geological Survey of Israel, Jerusalem, 95501 Israel

**Keywords:** Phosphate deposition, Sulfate-reducing bacteria, Sulfide-oxidizing bacteria, Lipid biomarkers, Cretaceous, Ghareb formation, Negev/Israel

## Abstract

**Background:**

On Late Cretaceous Tethyan upwelling sediments from the Mishash/Ghareb Formation (Negev, Israel), bulk geochemical and biomarker analyses were performed to explain the high proportion of phosphates in the lower part and of organic matter (OM) preserved in upper parts of the studied section. The profile is composed of three facies types; the underlying Phosphate Member (PM), the Oil Shale Member (OSM) and the overlying Marl Member (MM).

**Results:**

Total organic carbon (TOC) contents are highly variable over the whole profile reaching from 0.6% in the MM, to 24.5% in the OSM. Total iron (TFe) varies from 0.1% in the PM to 3.3% in the OSM. Total sulfur (TS) ranges between 0.1% in the MM and 3.4% in the OSM, resulting in a high C/S ratio of 6.5 in the OSM section. A mean proportion of 11.5% total phosphorus (TP) in the PM changed abruptly with the facies to a mean value of only 0.9% in the OSM and the MM.

The TOC/TOC_OR_ ratios argue for a high bacterial sulfate reduction activity and in addition, results from fatty acid analyses indicate that the activity of sulfide-oxidizing activity of bacteria was high during deposition of the PM, while decreasing during the deposition of the OSM.

**Conclusions:**

The upwelling conditions effected a high primary productivity and consequently the presence of abundant OM. This, in combination with high sulfate availability in the sediments of the PM resulted in a higher sulfide production due to the activity of sulfate-reducing bacteria. Iron availability was a limiting factor during the deposition of the whole section, affecting the incorporation of S into OM. This resulted in the preservation of a substantial part of OM against microbial degradation due to naturally-occurring sulfurization processes expressed by the high C/S ratio of 6.5 in the OSM.

Further, the abundant sulfide in the pore water supported the growth of sulfide-oxidizing bacteria promoting the deposition of P, which amounted to as much as 15% in the PM. These conditions changed drastically from the PM to the OSM, resulting in a significant reduction of the apatite precipitation and a high concentration of reactive S species reacting with the OM.

## Background

In most anoxic environments, hydrogen sulfide (H_2_S) originating from sulfate reduction will preferentially react with reduced iron (Fe) to form Fe monosulfides, which are transformed during diagenesis to pyrite [1]. However, when the supply of reduced Fe in the water column and pore water of the sediments is limited and organic matter (OM) is abundantly available, both sulfate reduction by one part of the OM and quenching of the reduced sulfur (S) species by the remaining part of the OM will occur, leading to formation of S-rich sedimentary OM [2,3]. Such conditions are typical of highly productive upwelling systems on continental margins with restricted inputs of Fe from terrigenous systems [4].

Many studies have been carried out to elucidate the mechanism of S reactions with OM in sediments e.g. [5]. Bacterially-produced inorganic S species, like H_2_S, polysulfides or elemental S, are potential agents for the reaction with the OM e.g. [6-8]. Incorporation of elemental S and polysulfides into OM is known to occur in association with two types of chemolithotrophic organisms, the green and purple sulfur bacteria living in photic zone euxinia e.g. [9] and sulfide-oxidizing bacteria such as *Thiomargarita*, *Thioploca* and *Beggiatoa* living at sediment surfaces in upwelling areas e.g. [10-12]. Upon reaction of these S species with the biomass during early diagenesis, low amounts of S are able to preserve high amounts of OM from microbial degradation. Therefore, incorporation of reduced S into the humic matrix of anoxic sediments will result in an increase of the C/S ratios compared to sediments deposited under normal marine conditions [13,14].

Dinur, et al. [15] investigated oil shale samples from the Ghareb Formation from different sites in Israel and found that 50-95% of the total S was organically bound. More recently, Amrani, et al. [16] found a similar value (85%) for an immature organic-rich limestone from the Ghareb Formation containing Type-II-S kerogen. Upon hydrous pyrolysis of these samples the early released H_2_S formed secondary pyrite, which was isotopically 21‰ lighter than the bulk organic S, indicating that the system was not in equilibrium [16]. Large S-isotopic discrimination comparing S incorporated in the oil and in its source kerogen indicated more open system conditions [16], meaning that fresh sulfate was introduced while sulfate reduction proceeded [17].

Results from elemental analysis (C and S) of sediments from the Ghareb Formation, which have been reported in several studies before e.g. [18,19], are rather characteristic of freshwater sediments, despite the fact that deposition took place in a marine environment. Two different reasons were given to explain this discrepancy. The deposition of the Ghareb Formation took place under normal marine conditions and the H_2_S generated in the sediment escaped partly into the atmosphere [18]. This would imply that the water column was entirely euxinic. Bein, et al. [19] provided a different explanation by proposing that deposition of the oil shale took place in the marine environment under a limited flux of sulfate into the sediment, which served to limit the availability of sulfate for OM consumption during sulfate reduction.

More recently, the importance of the chemolithotrophic bacteria such as *Thiomargarita*, *Thioploca* and *Beggiatoa* for S cycling in upwelling environments was recognized [20,21]. These organisms, which obtain their energy from oxidizing sulfide by using either molecular oxygen or nitrate [22], accumulate elemental S in their biomass and are enriched particularly in anoxic sediments, characterized by a high rate of sulfate reduction [11,23].

In sediments from the Namibian shelf, large populations of the sulfide-oxidizing bacteria *Thiomargarita* were found which are also responsible for the accumulation of phosphorite in the sediments [24]. Recently, the accumulation and deposition of phosphate of a marine *Beggiatoa* strain was documented under laboratory conditions [25]. The investigation of shelf sediments from central Chile showed that *Thioploca* influenced the mineralization of P in costal upwelling systems [26]. A close relationship between phosphorite formation and sulfate-reducing and sulfide-oxidizing bacteria has also been shown for other phosphogenic sediments, phosphatic laminites and phosphorites [11,12,24]. The greater availability of P in coastal upwelling regions has been associated with their higher biological productivity [27]. Modern phosphogenesis is predominantly located in the upwelling regions off Namibia, Chile, Peru, in the Gulf of California and the Arabian Sea [28] under suboxic to anoxic conditions.

The biomarker pattern of the sulfide-oxidizing bacteria is not very specific (mono-unsaturated n-C_16_ and n-C_18_ fatty acids are dominant) and, as such, it is difficult to trace back their presence in ancient sediments [11,12].

In the present study, the proportions of several elements (C, S, Fe and P) and the fatty acid composition were determined in Late Cretaceous sediments from the Mishash/Ghareb Formation (Negev/Israel), covering the facies types of phosphate, oil shale and marls to distinguish between the different depositional environments and explain the facies changes in the upwelling system.

## Material and methods

### Location and samples

During the Late Cretaceous, a highly productive upwelling regime existed along the southern margins of the Tethys, lasting nearly 20 million years (Myr) from the Santonian to the late Maastrichtian [29]. The sampled sedimentary profile was deposited during the late Campanian to the early Maastrichtian (71.6 to 69.85 Ma). The sediments, consisting of organic-rich carbonates and phosphorites, belong to the Upper Cretaceous-Eocene phosphate belt, which extends from South America (Colombia) over North Africa to the Middle East [29,30].

**Figure 1:**
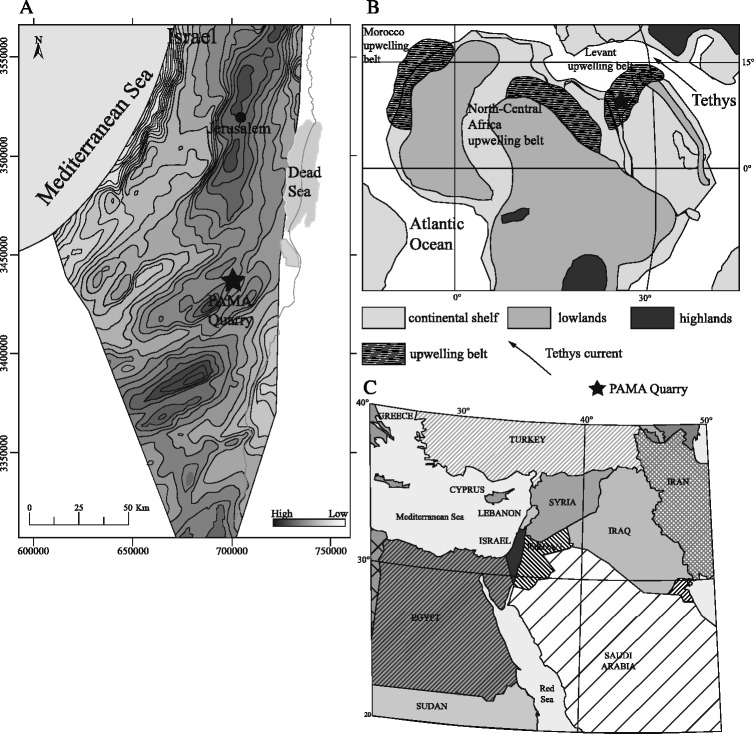
**Location map for the studied area. A)** Position of the PAMA Quarry (Efe Syncline) in a local map of Israel (UTM Coordinate system). Base topography is given in a structural map showing Syrian Arc structures (modified after Gardosh, et al. [74] and Almogi-Labin, et al. [75]). **B)** Inferred position of the PAMA Quarry within a paleogeographical map showing the upwelling belts that developed along the southern Tethys margin during the late Campanian and early Maastrichtian (from Ashckenazi-Polivoda, et al. [31]). **C)** Location of Israel shown on a political map of the Middle-East.

The samples were obtained from a fresh section within the PAMA quarry at Mishor Rotem in the northern Negev, Israel (Efe Syncline; 31°04′51.82″N; 35°10′02.85″E). The samples, unaltered by weathering, were obtained during mining activities. The sampling site is shown on a local map of Israel with topographical information (Figure 1A) and a paleogeographical reconstruction shows the settings during the Cretaceous (Figure 1B).

**Figure 2:**
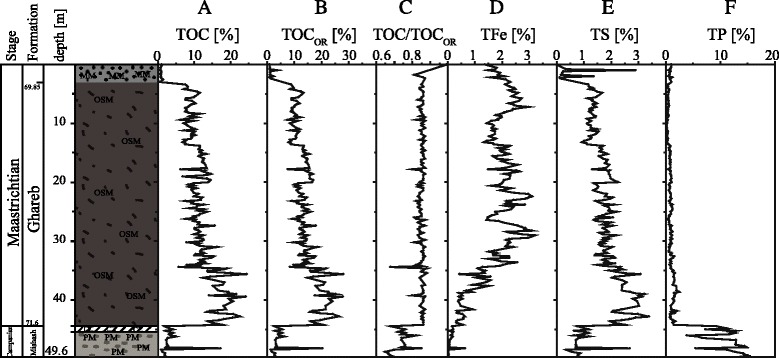
**Stratigraphy, age, depth and lithological profile (cf. Ashckenazi-Polivoda, et al.** **[** 31 **]): MM, Marl Member; OSM, oil shale member; CL, condensed layer; PM, Phosphate Member) of the sampled section with (A) variation in TOC, (B) calculated original TOC (TOC**_**OR**_ **), (C) TOC/TOC**_**OR**_ **ratio, (D) total Fe (TFe), (E) total S (TS) and (F) total P (TP).**

The sampled profile (Figure 2) of ca. 50 m is composed of a short sequence of the Mishash Formation [phosphate member (PM) 5.6 m] and the Ghareb Formation, mostly belonging to the oil shale member (OSM, 42 m) and a short sequence of the overlying marl member (MM, ca. 3 m).

The brownish-gray PM is mainly composed of peloids, bone fragments and fecal pellets [31,32]. The condensed layer consists of approximately 20% dolomite, 45% fluorapatite, 30% calcite, and 1–2 wt.% TOC, previously reported in Bein, et al. [19]. The OSM is composed of dark gray organic-rich carbonate mudstone with decreasing TOC content from the base to the top. The MM is a light-yellow marly chalk with TOC values below 1.0 wt.%. The lithology of the whole section has been described in detail by Ashckenazi-Polivoda, et al. [31] and Meilijson, et al. [32].

The TOC, total S (TS), total Fe (TFe) and total P (TP) values were determined on up to 211 samples of the profile.

### Sample preparation and analysis

For the measurement of TOC, the samples were analyzed using a LECO RC-412 carbon analyzer. Total Carbon (TC) was determined by combustion of the untreated sample material and TOC was determined after removing carbonate by acidification with hydrochloric acid (10%). The instrument was calibrated with the synthetic carbon standard 502–029 from LECO Corp. and an in-house CaCO_3_ standard. Approximately 100 mg of finely ground sample (<200 μm) were weighed into a quartz glass vessel before combustion.

The TS, TFe and TP contents were determined by means of energy dispersive X-ray fluorescence spectrometry (ED-XRF) on a selected set of samples (ca. every 0.2 m; 197 samples) at the Institute of Mineralogy and Geochemistry of the Karlsruhe Institute of Technology, Germany. For this, ca. 5 g of powdered sample material (<63 μm) was loaded into a Spectro-cup before sealing with 6 μm Mylar film. As radiation source, the instrument (Epsilon 5, PANalytical) used a tungsten X-ray tube, whereas detection and quantification was carried out with a Ge-detector. Detection limit for S was 100 mg kg^−1^. Analytical precision, based on repeated measurement of reference materials (AGV-1P, GXR-2, GXR-3, GXR-4,GXR-5, GXR-6, SCO-1, SDO-1P, SCO-1, SL1, SOIL V, SOIL VII) was generally better than ±5% for S and P and ±0.6% for TFe content (expressed as Fe_2_O_3_), respectively.

The TOC and TS values were used to calculate the TOC_OR_. The TOC_OR_ can be used for the estimation of the TOC before the loss of OM via sulfate reduction during diagenesis. It is calculated from Eq. 1 according to Vető, et al. [33].

1 $$ \mathrm{T}\mathrm{O}{\mathrm{C}}_{\mathrm{OR}} = \mathrm{T}\mathrm{O}\mathrm{C} + \mathrm{T}\mathrm{O}{\mathrm{C}}_{\mathrm{SR}} $$

TOC_SR_ is the loss of TOC by sulfate reduction and has been calculated using Eq. 2 [33]:

2 $$ \mathrm{T}\mathrm{O}{\mathrm{C}}_{\mathrm{SR}} = \mathrm{T}\mathrm{S}\bullet 0.75\bullet 1/\left(1\hbox{-} \mathrm{q}{\mathrm{H}}_2\mathrm{S}\right) $$

The value qH_2_S describes the re-oxidation of H_2_S by sulfide-oxidizing bacteria or its escape. With a value of 0.35, an intense sulfide re-oxidation by chemolithotrophic bacteria such as *Thioploca* spp. is assumed [34].

Bacterial sulfate reduction can be simplified described by Eq. 3. A reduction of 1% of S is equivalent to a loss of 0.75% of TOC.

3 $$ \mathrm{S}{{\mathrm{O}}_4}^{2\hbox{-} } + 2\ \mathrm{C}{\mathrm{H}}_2\mathrm{O}\kern0.65em \to \kern0.65em 2\ \mathrm{H}\mathrm{C}{{\mathrm{O}}_3}^{\hbox{-} } + {\mathrm{H}}_2\mathrm{S} $$

For the analysis of lipid biomarkers 20 g dried ground rock sample (<200 μm) were Soxhlet extracted for 30 h with dichloromethane/methanol (DCM/MeOH) 9:1. The solvent was evaporated using a rotary evaporator. An aliquot (10 mg) of the extract was separated into fractions of saturated hydrocarbons, aromatic hydrocarbons and polar N-, S-, O-containing compounds via column chromatography (20 g silica gel 60, Merck; glass column, 15 mm i.d). The composition of saturated and aromatic hydrocarbon fractions will not be discussed in the present paper. The fatty acids in the polar fractions were derivatized to trimethylsilylesters using 35 μl N,O-bis(trimethylsilyl)trifluoracet-amide (BSTFA) and 7 μl pyridine at 70°C for 1 h. The polar fractions were analyzed using gas chromatography–mass spectrometry (GC-MS) with a Trace GC Ultra gas chromatograph coupled to a dual stage quadrupole (DSQ II) mass spectrometer (Thermo Fisher Scientific). The system was equipped with a TR5-MS column (30 m, 0.25 mm i.d., 0.25 μm film thickness; Thermo Fisher Scientific). The diluted fractions were injected in splitless mode with a splitless time of 1 min. The GC oven temperature was programmed from 40 to 320°C at 3°C min^−1^.

The mass spectrometer was operated in electron ionization (EI, 70 eV) and full scan mode. Helium was used as carrier gas. The data were recorded, processed and used for quantitation of individual compounds with Xcalibur® software. As internal standard 3 μl of 1,1-binaphthyl (1 μg μ1^−1^) was added to the polar fraction.

## Results and discussion

### TOC, TFe, TS and TP

TOC was determined in 211 samples and the data are shown in Figure 2A. Within the PM, the mean value of TOC was 2.7%. At 48.2 m a thin section with a very high TOC value of 17.2% is intercalated (Figure 2A). The transition from the Ghareb Formation to the Mishash Formation is represented by a substantial hiatus visible as a condensed interval of 1 m thickness, which separates the PM from the OSM. A sharp increase in TOC from 1.9% to 17.7% is apparent between the condensed layer and OSM. Within the OSM, the TOC values tend to decrease from the bottom to the top of the section. In the lower part of the OSM section (42.8-35 m), the mean TOC values tends to decrease from 18.0% at the bottom to 10.3% at the top. One exceptional high value is observed at 35.6 m depth (24.5%). The facies change from the OSM to the MM is characterized by a sharp decrease of the TOC to 1.4%. In the MM section the TOC values are in a range between 0.6 to 1.4% (Figure 2A).

The TOC/TOC_OR_ ratio reflects the proportion of remaining TOC after bacterial sulfate reduction. The original TOC (TOC_OR_) and the TOC/TOC_OR_ ratios are plotted in Figure 2B and C together with the contents of TFe (Figure 2D), TS (Figure 2E) and TP (Figure 2F) determined in 197 samples. The TOC_OR_ (Figure 2B) varies largely parallel with the TOC content along the total profile. The lowest TOC/TOC_OR_ ratios, with a mean value of 0.72, are observed in the PM, indicative of a higher degree of sulfate reduction here compared to the OSM and MM sections. In the OSM, the TOC/TOC_OR_ is very homogeneous, with values varying between 0.68 and 0.88 (mean 0.85), implying stable environmental conditions during sedimentation. Furthermore, this indicates an intensive bacterial consumption of the originally deposited OM for sulfate reduction during deposition of most parts of the OSM. Similar reduction rates were reported from the recent upwelling system in the northeastern Arabian Sea [35]. In the MM the ratio of TOC/TOC_OR_ (Figure 2C) varies between 0.75 and 0.99, with high fluctuation indicating changing environmental conditions. The effect of sulfate reduction on the precipitation of phosphate is discussed later.

The TFe content along the profile shows a trend largely opposite to that observed for TOC (Figure 2D). The lowest abundance is in the PM (between 0.1% and 0.2%), interrupted by only one sharp maximum of 0.7% at 48.2 m. Within the hiatus, the TFe content increases from 0.1 to 0.6%. The TFe content varies between 0.5 and 3.1% for the OSM and between 1.4 and 2.4 for the MM, demonstrating a slightly increasing trend in concentrations towards the OSM.

Within the PM, the TS content (Figure 2E) fluctuates slightly around a mean value of 0.8%, with the exception of one sample at 48.2 m, which also shows a maximum in TOC and TFe content. At the transition to the adjacent OSM, the TS content increases significantly from 1.1 to 2.5%. The TS values vary somewhat in parallel with the TOC values. Within the OSM it follows the same trend as observed for TOC. The proportion of TS is higher in the lower part of the profile (mean value 2.6%) compared to the upper part (mean value 1.5). The TS content in the MM fluctuates between 0.1 and 2.9%, with two high outlier values at 1 m (2.9%) and 2 m (1.4%).

TP is significantly enriched in the PM and fluctuates between 3.9% and 15.0% with a mean proportion of 11.5% (Figure 2F). With the facies change from the PM/hiatus to the OSM the proportion of TP in the sediments decreases drastically. Further upwards along the profile, the amount of TP is low with a mean value of only 0.9 in the OSM and the MM.

### Correlation of TOC and TS

**Figure 3:**
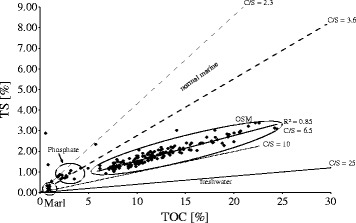
**Plot of TS vs. TOC (according to Berner and Raiswell** **[** 36 **]** **with modifications adopted from Leventhal** **[** 37 **]** **).** The fine dotted line indicates non-marine (freshwater) environments of deposition. Data points from normal marine environments usually plot between both dashed lines. The samples from the MM and the PM plot along the line indicating normal marine conditions, whereas data points of the OSM plot in the field between normal marine and freshwater conditions.

Figure 3 shows the correlation diagram of TOC and TS, which was previously introduced by Berner and Raiswell [36] and Leventhal [37]. The dashed lines indicate the field for sediments deposited under normal marine conditions, with C/S values between 2.8 [36] and 3.6 [37]. C/S values for freshwater sediments will plot close to the dotted lines, with values between 10 [38] and 25 [37]. As expected, the values obtained for samples from the three different facies types plot in different areas of the diagram. The values from the PM plot between the lines of 2.8 and 3.6, suggestive of normal marine conditions [38].

These results are in contrast to organic geochemical and micropaleontological investigations of this section. According to the very low pristane/phytane ratio [39] and investigations based on the foraminifera assemblage [31], this part of the sequence should have been deposited under oxygen deficient conditions. These conditions would imply a higher TOC content of the sediment and hence resulting in a higher C/S ratio. This discrepancy in the C/S ratio may be attributed to an intensive bacterial decomposition of the OM, due to sulfate reduction and sulfide oxidation, in combination with the deposition of phosphate in the PM e.g. [12,24,40-42].

The TS values for the OSM correlate strongly (R^2^ = 0.85) with TOC values and provide a slope of 0.12. In general, this is a characteristic value for euxinic environments [18]. However, biomarkers such as aryl isoprenoids, indicating photic zone euxinia were absent in the GC-MS analyses of all sediment extracts. The samples from the OSM plot between the freshwater and normal marine field and had a mean C/S ratio of 6.5. Similar C/S ratios have previously been reported for samples from the Ghareb and the Mishash Formation in Mishor Rotem [18] and for core samples from the Zin Valley and Shefela [19]. This reflects the higher primary productivity [29] and preservation of the OM during deposition of this section.

C/S ratios previously found in sediments from the Benguela, Peru and Oman upwelling zones are 1.3, 1.8 and sometimes even 2.8 times higher, compared to C/S ratios determined from sediments deposited under normal marine conditions [43]. This provides further evidence for an enhanced preservation of OM in upwelling environments, due to naturally occurring sulfurization processes. The C/S ratio of the OSM in the present study is between that of the Peru and Oman upwelling system (2.3 times higher than expected for normal marine conditions as shown in Figure 3). Comparable results with C/S values of more than twice as high as expected from normal marine sediments were also reported for marine upwelling sediments from the Arabian Sea [35,44].

The low amount of TFe, particularly in the PM and the lower section of OSM (Figure 2D), also supports that a significant proportion of TS has been fixed in the organic matrix, due to the limited Fe availability in the depositional environment. The OM becomes more resistant against microbial degradation, whereupon high amounts of organic carbon can be preserved by low amounts of reduced S [43,45]. This was previously attributed to a low temperature vulcanization process, in which macromolecular organic compounds are generated through sulfide bridges, preserving the OM in environments where reduced S is not rapidly consumed by Fe^2+^ in the water column or pore water [6]. This is well reflected by the geochemical data in Figure 2 showing an inverse trend for S/C and Fe in the OSM.

This is largely consistent with the previously reported proportion of 85% organically bound S in the Ghareb Formation [16]. Consequential, OSM samples with ca. 15% TOC contain about 2% organically bound S. This gives a TOC/S_org_ ratio of 7.5, which can be compared with TOC/S_org_ values for kerogen form the Peruvian upwelling system (6.6 to 33; Mossmann, et al. [46]) and from the Monterey oil shale (15 to 50; Orr [47]). The degree of OM sulfurization in the Mishash/Ghareb Formation therefore tends to be slightly higher than in recent sediments from the Peruvian upwelling area and much higher than in the Miocene Monterey Formation, which was also deposited in an upwelling environment.

However, the excess H_2_S in Fe-limited environments might not only react with organic compounds but might also be re-oxidized. According to Goldhaber, et al. [48] and Jørgensen [49], up to 90% of the H_2_S produced in the shelf sediments with concomitant high rates of bioproduction and high bioturbation might be lost due to re-oxidation after transport to the surface.

### Correlation of TOC, TS and TFe

**Figure 4:**
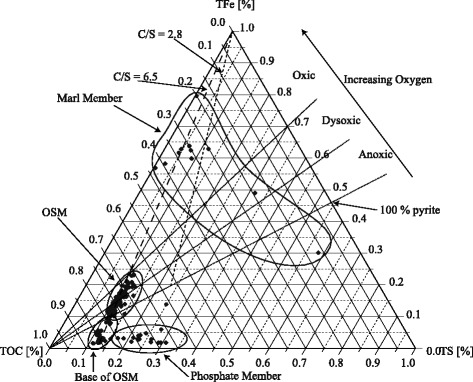
**Ternary plot of iron, sulfur and organic carbon according to Dean and Arthur** **[** 50 **]** **.** The fine dashed line represents a C/S ratio of 2.8 which is indicative for samples deposited under normal marine conditions [36]. The coarse dashed line results from the measured data on samples from the OSM with a mean C/S ratio of 6.5. The different facies types are marked with elliptical labels. The OSM samples are separated in an upper part of the section labeled as OSM and a lower part of the section labeled “Base of OSM”. The fine dotted line (100% pyrite) represents conditions where all reactive iron in the sediment would be present as pyrite. With fine dotted lines different oxygenation levels are marked according to Ross and Bustin [51].

Figure 4 shows a ternary correlation diagram for TFe, TS and TOC, originally introduced by Dean and Arthur [50] for the reconstruction of paleoenvironmental conditions during deposition of sediments. Fine dotted lines were used to illustrate environments of sediment deposition under oxic, dysoxic and anoxic conditions [51]. Samples plotting below the line of 100% Fe fixation as pyrite are characterized by an excess of S and the presence of organically bound S compounds (OSC) [50,52]. The data from the MM samples plot with two exceptions in the field of oxic conditions, which is consistent with the low amount of organic carbon in this section. In contrast, data from the OSM samples argue for deposition under dysoxic to anoxic conditions (see Figure 4). Samples from the base of the OSM plot in the field indicating excess of S during sedimentation. This is consistent with the observations from the analysis of the pristane/phytane ratio [39] and micropaleontological investigations [31] of this section indicating a low sea floor aeration.

Also included in Figure 4 is the empirically found relationship between TOC and TS for sediments deposited under “normal” marine conditions [36], shown by the fine dashed line indicating a C/S ratio of 2.8. For the three different facies types three different trends in the diagram are apparent. The samples from the PM show a fluctuating C/S ratio between 2.8 and 6.5. The PM samples clearly plot in the field below the pyrite line, indicating Fe limitation and an excess of S availability. OM is still available, but in highly biodegraded form, as indicated by results from GC-MS analysis of the saturated hydrocarbon fraction of samples from those sections. Short to long chain n-alkanes in the PM section are completely degraded, while in the OSM section these n-alkanes are present.

In accordance with Dean and Arthur [50], the mean C/S ratio of 6.5 in the OSM, indicated by the coarse dashed line, suggests that a high proportion of S is not present as pyrite. Similar values were found by Lückge, et al. [53] in samples from the Oman upwelling region, likely due to an increased incorporation of S into OM as a result of iron-limiting conditions. An abundance of reactive OM is associated with highly productive upwelling systems, which increases the rate of sulfate reduction to H_2_S [36]. This, in combination with a limited incursion of Fe increases the amount of organically bound S.

In the ternary diagram (Figure 4), the OSM samples neither plot along the 100% pyrite line nor along the C/S 2.8 line; instead the data points crosscut the 100% pyrite line, with samples from the OSM base plotting below, and samples from the OSM top plotting above this line. However, this cannot be interpreted as a result of changing environmental conditions from Fe-limited conditions to OM-limited conditions during deposition of the sediments going from the bottom to the top of the profile. Instead, Fe-limited conditions prevailed throughout deposition of the whole section but were more apparent during deposition of the bottom part of the OSM compared to the upper part. This particular situation will develop when the highly abundant OM in upwelling sediments is degraded by sulfate-reducing bacteria and more H_2_S is generated than can be quenched by the reduced Fe in the sediment. In this case, refractory sulfurized OM is produced, which cannot be utilized by sulfate-reducing bacteria. A similar situation has been found in late Albian samples from the North Atlantic studied by Hofmann, et al. [54], by way of Fe-S-TOC relationships comparable to those found in the OSM facies.

The samples from the MM also show high variability in the C/S ratio. A mean C/S ratio cannot be derived from these data. With one exception, the samples plot above the pyrite line, which indicates restricted availability of degradable OM during sediment deposition. This was probably effected on the one hand by the reduced productivity in the upwelling system and on the other hand by an increasing sea floor aeration [31]. Such OM- limited conditions were previously described by Hofmann, et al. [54] from the western North Atlantic drilling Site 386.

### Correlation of phosphate precipitation with sulfate reduction

In recent publications it was demonstrated that sulfate-reducing and sulfide-oxidizing bacteria played a crucial role in the precipitation of phosphate in ancient and modern upwelling systems e.g. [12,24,25,40,41,55-57].

**Figure 5:**
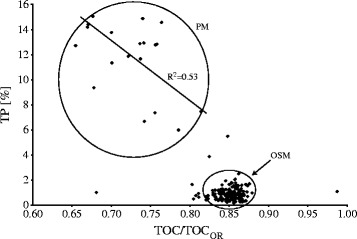
**Correlation diagram of TP[%] and TOC/TOC**_**OR**_ **ratio.** The data points belonging to the OSM and PM are marked. The sulfate reduction and proportion of TP show a strong correlation (R^2^ = 0.53).

Figure 5 shows the correlation of the TOC/TOC_OR_ ratio and the proportion of P [%] according to Lückge, et al. [35]. The correlation of TOC/TOC_OR_ and TP was used to determine whether the phosphate deposition was influenced by the sulfate-reducing process.

This close relation between the precipitation of phosphate and the reduction of sulfate is reflected by the geochemical data of the analyzed profile. The mean TOC/TOC_OR_ ratio of 0.73 in the PM section indicates an intensive sulfate-reducing process and is in accordance with the accumulation of TP up to 15% (Figure 2C and F). The PM and OSM sections were analyzed separately because of the clearly different environmental conditions which prevailed during their sedimentation. In contrast to the OSM section, the phosphates in the PM segment significantly correlate with the TOC/TOC_OR_ ratio (R^2^ = 0.53).

Further support for the interaction between sulfide oxidation and phosphate precipitation comes from Schulz and Schulz [24], who analyzed Namibian shelf sediments, populated by the sulfide-oxidizing bacterium *Thiomargarita*. They found a correlation between the occurrence of these sulfide-oxidizing bacteria and increased deposition of phosphate. Goldhammer, et al. [10] used organic rich sediments from the Benguela upwelling system to analyze the role of sulfide-oxidizing bacteria in the sequestration of P. With ^33^P radiotracer the accumulation of P in these bacteria and the release of inorganic phosphate under anoxic conditions, from which apatite can precipitate, was observed. Goldhammer, et al. [10] found that the large sulfide-oxidizing bacteria gain their energy from nitrate which directly influences the precipitation of P under anoxic conditions.

**Figure 6:**
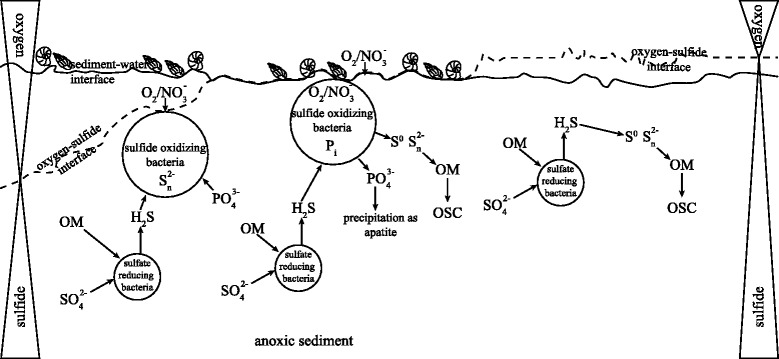
**Schematic illustration of bacterial sulfate reduction and sulfide oxidation under changing redox conditions and the effect on the phosphate deposition (Modified after Brock and Schulz-Vogt** **[** 25 **] and Dale, et al.** **[** 42 **]).**

Brock and Schulz-Vogt [25] cultivated sulfide-oxidizing bacteria of the species *Beggiatoa* and demonstrated that these bacteria store large amounts of phosphate under oxic conditions. These bacteria live at the oxygen-sulfide interface of the sediment [22]. The sulfide is produced in the deeper sediment by bacteria reducing sulfate and oxidizing organic material to gain their energy. The large sulfide-oxidizing bacteria use oxygen or nitrate to re-oxidize the sulfide to sulfate and gain energy out of this process [12,22]. The nitrate is stored in vacuoles and is used under the euxinic conditions in the sediment for the oxidation of sulfide [22]. Under conditions of low sulfide concentrations, bacteria like *Beggiatoa* store polyphosphates in vacuoles within their cells e.g. [12,25,58]. When switching from oxic to anoxic conditions with high sulfide concentrations, the stored polyphosphate is hydrolyzed by these bacteria and phosphate is released to the sediment and precipitates as apatite [25] (Figure 6).

Figure 6 illustrates the link between the interaction of sulfate reduction, sulfide oxidation and phosphate precipitation during the deposition of the PM and the OSM.

The left side of Figure 6 shows the conditions that prevail in Fe-poor sediments when the oxygen-sulfide interface is below the sediment-water interface. In this case the availability of sulfate for sulfate-reducing bacteria is low since diffusion of sulfate from the sea water into the pore water of the sediment is hindered. As a consequence, H_2_S production and the activity of sulfide-oxidizing bacteria will also be low due to the restricted availability of oxygen and nitrate deeper in the sediment. Under these conditions sulfide-oxidizing bacteria re-oxidize H_2_S to sulfate will take up phosphate and accumulate polyphosphate in their cell walls for energy storage e.g. [10,24,25]. Abundant benthic foraminifera will colonize the sediment-water interface. When the oxygen-sulfide interface interferes with the sediment-water interface, the situation shown in the middle of Figure 6 will establish. More sulfate will be available in the sediment and sulfate reduction will increase. High sulfide concentrations in the sediments will force the sulfide-oxidizing bacteria to release S and polysulfides as well as phosphate into the sediment [59] and the phosphate will be precipitated as apatite. Benthic foraminifera can survive under these conditions and will co-exist with sulfide-oxidizing bacteria [60]. The elemental S and the polysulfides will react with OM in the sediment under formation of OSC [61-63]. When the oxygen-sulfide interface is moving further upwards into the water column (Figure 6 right side) sulfide-oxidizing bacteria cannot survive in the sediment due to the absence of molecular oxygen. The H_2_S from sulfate reduction generates sulfidic bottom water conditions and accounts for the increasing amounts of OSC generated in the Fe-poor depositional environment.

During deposition of the PM the situation shown on the left side and in the middle of Figure 6 prevailed with the oxygen-sulfide interface moving temporarily from the surface into the sediment which is in accordance with the high abundance of benthic foraminifera [31]. Under these oscillating redox conditions the sulfide-oxidizing bacteria have their greatest phosphate storing ability [42]. This forced an accumulation of P, which is 7 to 10 times higher in concentration compared to the OSM.

During deposition of the OSM the oxygen sulfide interface remained close to the sediment interface and the abundance of both sulfide-oxidizing bacteria and benthic foraminifera decreased. The data obtained in the present study do not support the establishment of an euxinic water column during deposition of the OSM (Figure 6 right side) since benthic foraminifera are still present throughout the whole section although in lower abundance compared to the PM. Within the OSM sea floor aeration was lower in the bottom section and increased towards the top of the OSM section and even further in the MM section according the composition of planktic and benthic foraminifera [31].

The elevated primary productivity of the upwelling system supports the development of an OMZ in the mean water [29]. During the deposition of the OSM, conditions in the sediment became anoxic due to the expansion of the OMZ to the sediment-water interface, as indicated by the accumulation of OM and the high abundance of organically bound S (up to 90%) in this section [15,19]. The more anoxic and sulfidic conditions during the sedimentation of the OSM lead to more resistant OM being preserved as organic S compounds (OSC). Various of these compounds like thiophenes, thiolanes, hopanoid thiophenes and further compounds originating from these environmental conditions were detected by our GC-MS analyses. The diagenetic pathways and conditions of their origin were in detail described previously from the geologically corresponding Jurf ed Darawish sediments in Jordan e.g. [45,64-67].

During deposition of the OSM the large sulfide-oxidizing bacteria decreased in abundance compared to the PM and were not further able to accumulate polyphosphate for energy storage. The deposition of P would no longer be promoted by these bacteria. This is reflected by the comparably low amounts of P (ca. 1%) in this section, values which are common for marine sediments [68].

Soudry and Champetier [69] analyzed Campanian phosphorite sediments from the Negev/Israel with microscopic and SEM techniques. They found filamentous, tubular structures in the phosphatic matrix and suggested an algal or cyanobacterial origin. However, similar structures are also known from sulfur bacteria like *Beggiatoa* and *Thioploca* living in upwelling environments of the modern oceans [70] and it seems very likely that the intertwined filamentous structures described by Soudry and Champetier [69] derive from sulfide-oxidizing bacteria like *Beggiatoa* or *Thioploca*. In recent studies the sulfide-oxidizing bacteria were described as colorless filaments [11,22,24,57].

More recently, Soudry, et al. [71] analyzed sediments from the Negev of Mid-Cretaceous to Eocene age and demonstrated that the P accumulation rates reach their maximum during the Campanian/Maastrichtian, which is in agreement with our data. The major source of the P was located in the Northern Pacific ocean indicated by the analysis of Nd isotopes [71].

### Fatty acid analysis

The analysis of the polar fraction after derivatization was further used to provide evidence for the influence of sulfate-reducing and sulfide-oxidizing bacteria in the sediments of the former upwelling system. The polar fraction of the sediment extracts contains fatty acids in the range from C_9_ to C_30_. They are mostly the dominating compound class of the polar fraction. Over the whole profile, several samples show a sigmoidal distribution of the fatty acids with a first maximum at C_16:0_ or C_18:0_ and a second at C_24:0_. In most cases the C_16:0_ and C_18:0_ fatty acids are the dominating compounds, although long chain fatty acids (C_20_-C_30_) show a greater resistance towards degradation [11]. Generally, the even numbered fatty acids predominate. The longest of these fatty acids (C_26_-C_30_) were present in low to trace amounts and could be determined in a few samples over the whole profile. This further supports the previous suggestion of a minor contribution of terrestrial OM to the upwelling sediments derived from the low total organic nitrogen (TON) content and high C/N ratios [39]. The C_24:0_ fatty acid shows the highest abundance among the long chain fatty acids. The source of this fatty acid is widespread in marine sediments and likely originates from phytoplankton in upwelling environments, as other, especially land plant derived biomarkers are missing.

**Figure 7:**
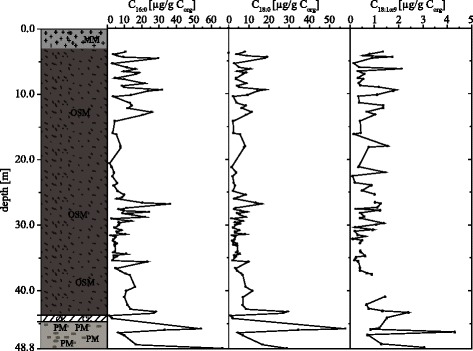
**Distribution of the saturated fatty acids C**_**16:0**_ **and C**_**18:0**_ **and the monounsaturated fatty acid C**_**18:1ω9**_ **over the profile. Concentrations are shown in μg/g C**_**org**_ **.**

The most dominating components in the polar fraction are the n-C_16:0_ and n-C_18:0_ fatty acids with highest amounts of 67 μg/g C_org_ and 57 μg/g C_org_ determined in the PM section (Figure 7). The abundant appearance of these lipids reflects the phytoplanktonic origin and the very high productivity in the upwelling system during the sedimentation of the PM. This is supported by the results of micropaleontological analyses on the foraminiferal assemblages [29,31], whereupon the high productivity was derived from the planktonic foraminifers. Recently, the molecular biomarker and elemental composition of phosphorites from the Miocene Monterey Formation were investigated [41]. In accordance with our results, the fatty acid composition was also dominated by C_16:0_ and C_18:0_ fatty acids [41]. Further support comes from a recent study of the fatty acid composition from sediments of the Chilean coastal upwelling [72]. The analyzed sediments showed a comparable composition of fatty acids and a predominance of the C_16:0_ [72].

Trace amounts of the branched fatty acid *i*-C_15:0_ determined in several samples of the PM and OSM sections indicate the expected activity of sulfate-reducing bacteria during the sedimentation. The occurrence of this branched fatty acid in phosphatic laminites from *Beggiatoa* populated sediments was previously reported from the Peru upwelling [12] and from phosphorites from the Miocene Monterey Formation where their appearance was also explained with the occurrence of sulfate-reducing bacteria [41].This further supports our conclusion from the bulk geochemical data and the TOC/TOC_OR_ ratio that abundant OM was consumed by sulfate-reducing bacteria. Additionally, it supports the co-occurrence of phosphate deposition and sulfate-reducing and sulfide-oxidizing processes as reported in previous studies [11,12,24,25].

Moreover, the detection of the monounsaturated fatty acids oleic acid (C_18:1*ω*9_) and vaccenic acid (C_18:1*ω*7_), which wereboth detected over the whole profile except in the condensed layer (Figure 7), provides additional evidence that the sedimentation of the PM and OSM was influenced by *Beggiatoa* and *Thioploca*. These monounsaturated fatty acids were previously reported from the *Beggiatoa* and *Thioploca* populated sediments from the upwelling regions off Namibia, Peru and Chile [11,73] and recently from the Miocene Monterey Formation [41]. Highest concentrations of these substances in the PM are in accordance with highest intensities of sulfate reduction derived from the geochemical data (Figures 2 and 7). Upwards in the profile, the concentration of these fatty acids decreased significantly.

Recent analysis of sediments from the Chilean upwelling system shows that the concentration of these monounsaturated fatty acids decreased significantly with depth, due to the habitat of these sulfur bacteria in the upper sediment level. The relatively low amounts of the detected unsaturated fatty acids are possibly due to their increased sensitivity towards degradation [72].

The analysis of the fatty acid composition of the PM and OSM samples supports the suggestion that these sediments were deposited under the influence of both sulfate-reducing bacteria and sulfide-oxidizing bacteria.

## Conclusions

The geochemical analyses of the phosphorites and oil shales from the Late Cretaceous Tethyan Ghareb and Mishash Formation provide evidence for a link between sulfate reduction, sulfide oxidation and phosphate accumulation during the sedimentation process. Changing environmental conditions influence the uptake and release of phosphate via sulfide-oxidizing bacteria. When anoxic conditions prevail deeper in the sediment, less sulfide will be available in the pore water and bacteria like *Beggiatoa* will store polyphosphates in vacuoles within their cells. When the oxygen-sulfide interface approaches the sediment-water interface, more oxygen and nitrate become available for sulfide-oxidizing bacteria, which then store large amounts of nitrate to further oxidize the sulfide produced by sulfate-reducing bacteria. This will cause the liberation of phosphate into the pore water followed by its precipitation as apatite.

The high abundance of benthic foraminifers present in the PM provides evidence that the oxygen-sulfide interface did not expand beyond the sediments into the water during the deposition of this section.

The oscillating redox conditions present during the deposition of the PM changed to permanently anoxic pore water conditions and the activity of sulfide-oxidizing bacteria became locally restricted to the area close to the sediment-water interface. An expansion of the anoxic zone over the upper zone of the sediment into the water column and displacement of the oxygen would result in the complete extinction of the sulfide-oxidizing bacteria. This did not happen for longer times during deposition of the OSM. The low abundance of Fe avoided the effective scavenge of sulfide and reduced S species played a crucial role in the sedimentation process.

The OM was the only available sink for the excess sulfide produced, resulting in an accumulation of S in the organic matrix and a preservation of this material against microbial degradation. This process, known as natural vulcanization, is mainly responsible for the high accumulation of organic material in the sediments of the OSM and the abundant appearance of organic S compounds.

## References

[1] Berner RA (1970). Sedimentary pyrite formation. Am J Sci.

[2] Hartgers WA, Lòpez JF, Sinninghe Damsté JS, Reiss C, Maxwell JR, Grimalt JO (1997). Sulfur-binding in recent environments: II. Speciation of sulfur and iron and implications for the occurrence of organo-sulfur compounds. Geochim Cosmochim Ac.

[3] Schaeffer P, Adam P, Philippe E, Trendel JM, Schmid J-C, Behrens A (2006). The wide diversity of hopanoid sulfides evidenced by the structural identification of several novel hopanoid series. Org Geochem.

[4] Eglinton TI, Repeta DJ, Holland HD, Turekian KK (2011). Organic matter in the contemporary ocean. Geochemistry of Earth Surface Systems: From the Treatise on Geochemistry.

[5] Werne JP, Hollander DJ, Behrens A, Schaeffer P, Albrecht P, Sinninghe Damsté JS (2000). Timing of early diagenetic sulfurization of organic matter: a precursor-product relationship in Holocene sediments of the anoxic Cariaco Basin, Venezuela. Geochim Cosmochim Ac.

[6] Adam P, Schmid JC, Mycke B, Strazielle C, Connan J, Huc A (1993). Structural investigation of nonpolar sulfur cross-linked macromolecules in petroleum. Geochim Cosmochim Ac.

[7] Aizenshtat Z, Krein EB, Vairavamurthy MA, Goldstein TP, Vairavamurthy MA, Schoonen MAA, Eglinton TI, Luther GW, Manowitz B (1995). Role of sulfur in the transformations of sedimentary organic matter: a mechanistic overview. Geochemical Transformations of Sedimentary Sulfur.

[8] Amrani A, Aizenshtat Z (2004). Mechanisms of sulfur introduction chemically controlled: δ^34^S imprint. Org Geochem.

[9] Tang K, Baskaran V, Nemati M (2009). Bacteria of the sulphur cycle: an overview of microbiology, biokinetics and their role in petroleum and mining industries. Biochem Eng J.

[10] Goldhammer T, Brüchert V, Ferdelman TG, Zabel M (2010). Microbial sequestration of phosphorus in anoxic upwelling sediments. Nature Geosci.

[11] Arning ET, Birgel D, Schulz-Vogt HN, Holmkvist L, Jørgensen BB, Larson A (2008). Lipid biomarker patterns of phosphogenic sediments from upwelling regions. Geomicrobiol J.

[12] Arning ET, Birgel D, Brunner B, Peckmann J (2009). Bacterial formation of phosphatic laminites off Peru. Geobiology.

[13] Nissenbaum A, Kaplan I (1972). Chemical and isotopic evidence for the in situ origin of marine humic substances. Limnol. Oceanog.

[14] Francois R (1987). A study of sulphur enrichment in the humic fraction of marine sediments during early diagenesis. Geochim Cosmochim Ac.

[15] Dinur D, Spiro B, Aizenshtat Z (1980). The distribution and isotopic composition of sulfur in organic-rich sedimentary rocks. Chem Geol.

[16] Amrani A, Lewan MD, Aizenshtat Z (2005). Stable sulfur isotope partitioning during simulated petroleum formation as determined by hydrous pyrolysis of Ghareb limestone, Israel. Geochim Cosmochim Ac.

[17] Schwarcz HP, Burnie SW (1973). Influence of sedimentary environments on sulfur isotope ratios in clastic rocks: a review. Miner Deposita.

[18] Minster T, Nathan Y, Raveh A (1992). Carbon and sulfur relationships in marine Senonian organic-rich, iron-poor sediments from Israel - a case study. Chem Geol.

[19] Bein A, Almogi-Labin A, Sass E (1990). Sulfur sinks and organic carbon relationships in Cretaceous organic-rich carbonates; implications for evaluation of oxygen-poor depositional environments. Am J Sci.

[20] Zopfi J, Böttcher ME, Jørgensen BB (2008). Biogeochemistry of sulfur and iron in *Thioploca*-colonized surface sediments in the upwelling area off central Chile. Geochim Cosmochim Ac.

[21] Sievert SM, Kiene RP, Schultz-Vogt HN (2007). The sulfur cycle. Oceanography.

[22] Schulz HN, Jørgensen BB (2001). Big bacteria. Annu Rev Microbiol.

[23] Schulz HM, Bechtel A, Sachsenhofer RF (2005). The birth of the Paratethys during the Early Oligocene: from Tethys to an ancient Black Sea analogue?. Global Planet Change.

[24] Schulz HN, Schulz HD (2005). Large sulfur bacteria and the formation of phosphorite. Science.

[25] Brock J, Schulz-Vogt HN (2011). Sulfide induces phosphate release from polyphosphate in cultures of a marine *Beggiatoa* strain. ISME J.

[26] Holmkvist L, Arning ET, Küster-Heins K, Vandieken V, Peckmann J, Zabel M (2010). Phosphate geochemistry, mineralization processes, and *Thioploca* distribution in shelf sediments off central Chile. Mar Geol.

[27] Ruttenberg KC. The global phosphorus cycle. In Treatise on Geochemistry, Edited by Holland HD, Turekian KK. Elsevier Science: Oxford; 2003.

[28] Föllmi KB (1996). The phosphorus cycle, phosphogenesis and marine phosphate-rich deposits. Earth-Sci Rev.

[29] Almogi-Labin A, Bein A, Sass E (1993). Late Cretaceous upwelling system along the southern Tethys margin (Israel): interrelationship between productivity, bottom water environments, and organic matter preservation. Paleoceanography.

[30] Pufahl PK, Grimm KA, Abed AM, Sadaqah RMY (2003). Upper Cretaceous (Campanian) phosphorites in Jordan: implications for the formation of a south Tethyan phosphorite giant. Sediment Geol.

[31] Ashckenazi-Polivoda S, Abramovich S, Almogi-Labin A, Schneider-Mor A, Feinstein S, Püttmann W (2011). Paleoenvironments of the latest Cretaceous oil shale sequence, southern Tethys, Israel, as an integral part of the prevailing upwelling system. Paleogeogr Paleoclimatol Paleoecol.

[32] Meilijson A, Ashckenazi-Polivoda S, Ron-Yankovich L, Illner P, Alsenz H, Speijer RP (2014). Chronostratigraphy of the Upper Cretaceous high productivity sequence of the southern Tethys, Israel. Cretaceous Res.

[33] Vető I, Hetényi M, Demény A, Hertelendi E (1994). Hydrogen index as reflecting intensity of sulphidic diagenesis in non-bioturbated, shaly sediments. Org Geochem.

[34] Ferdelman TG, Lee C, Pantoja S, Harder J, Bebout BM, Fossing H (1997). Sulfate reduction and methanogenesis in a *Thioploca*-dominated sediment off the coast of Chile. Geochim Cosmochim Ac.

[35] Lückge A, Ercegovac M, Strauss H, Littke R (1999). Early diagenetic alteration of organic matter by sulfate reduction in Quaternary sediments from the northeastern Arabian Sea. Mar Geol.

[36] Berner RA, Raiswell R (1983). Burial of organic carbon and pyrite sulfur in sediments over phanerozoic time: a new theory. Geochim Cosmochim Ac.

[37] Leventhal JS (1995). Carbon-sulfur plots to show diagenetic and epigenetic sulfidation in sediments. Geochim Cosmochim Ac.

[38] Berner RA, Raiswell R (1984). C/S method for distinguishing freshwater from marine sedimentary rocks. Geology.

[39] Schneider-Mor A, Alsenz H, Ashckenazi-Polivoda S, Illner P, Abramovich S, Feinstein S (2012). Paleoceanographic reconstruction of the Late Cretaceous oil shale of the Negev, Israel: integration of geochemical, and stable isotope records of the organic matter. Paleogeogr Paleoclimatol Paleoecol.

[40] Algeo TJ, Ingall E (2007). Sedimentary C_org_:P ratios, paleocean ventilation, and Phanerozoic atmospheric pO_2_. Paleogeogr Paleoclimatol Paleoecol.

[41] Berndmeyer C, Birgel D, Brunner B, Wehrmann LM, Jöns N, Bach W (2012). The influence of bacterial activity on phosphorite formation in the Miocene Monterey Formation, California. Paleogeogr Paleoclimatol Paleoecol.

[42] Dale AW, Bertics VJ, Treude T, Sommer S, Wallmann K (2013). Modeling benthic–pelagic nutrient exchange processes and porewater distributions in a seasonally hypoxic sediment: evidence for massive phosphate release by *Beggiatoa*?. Biogeosciences.

[43] Morse JW, Emeis KC (1990). Controls on C/S ratios in hemipelagic upwelling sediments. Am J Sci.

[44] Schenau SJ, Passier HF, Reichart GJ, de Lange GJ (2002). Sedimentary pyrite formation in the Arabian Sea. Mar Geol.

[45] Sinninghe Damsté JS, Rijpstra WIC, Kock-van Dalen AC, de Leeuw JW, Schenck PA (1989). Quenching of labile functionalised lipids by inorganic sulphur species: evidence for the formation of sedimentary organic sulphur compounds at the early stages of diagenesis. Geochim Cosmochim Ac.

[46] Mossmann JR, Aplin AC, Curtis CD, Coleman ML (1991). Geochemistry of inorganic and organic sulphur in organic-rich sediments from the Peru margin. Geochim Cosmochim Ac.

[47] Orr WL (1986). Kerogen/asphaltene/sulfur relationships in sulfur-rich Monterey oils. Org Geochem.

[48] Goldhaber MB, Aller RC, Cochran JK, Rosenfeld JK, Martens CS, Berner RA (1977). Sulfate reduction, diffusion, and bioturbation in Long Island Sound sediments; report of the FOAM group. Am J Sci.

[49] Jørgensen BB (1978). A comparison of methods for the quantification of bacterial sulfate reduction in coastal marine sediments. Geomicrobiol J.

[50] Dean WE, Arthur MA (1989). Iron-sulfur-carbon relationships in organic-carbon-rich sequences I: Cretaceous Western Interior Seaway. Am J Sci.

[51] Ross DJK, Bustin RM (2009). Investigating the use of sedimentary geochemical proxies for paleoenvironment interpretation of thermally mature organic-rich strata: examples from the Devonian–Mississippian shales, Western Canadian Sedimentary Basin. Chem Geol.

[52] Arthur MA, Sageman BB (1994). Marine black shales: depositional mechanisms and environments of ancient-deposits. Annu Rev Earth Pl Sci.

[53] Lückge A, Boussafir M, Lallier-Vergès E, Littke R (1996). Comparative study of organic matter preservation in immature sediments along the continental margins of Peru and Oman. Part I: results of petrographical and bulk geochemical data. Org Geochem.

[54] Hofmann P, Ricken W, Schwark L, Leythaeuser D (2000). Carbon-sulfur-iron relationships and ^13^C of organic matter for Late Albian sedimentary rocks from the North Atlantic Ocean: paleoceanographic implications. Paleogeogr Paleoclimatol Paleoecol.

[55] Arning ET, Lückge A, Breuer C, Gussone N, Birgel D, Peckmann J (2009). Genesis of phosphorite crusts off Peru. Mar Geol.

[56] Lepland A, Joosu L, Kirsimäe K, Prave AR, Romashkin AE, Črne AE (2014). Potential influence of sulphur bacteria on Palaeoproterozoic phosphogenesis. Nature Geosci.

[57] Brock J (2011). Impact of sulfide-oxidizing bacteria on the phosphorus cycle in marine sediments.

[58] Brock J, Rhiel E, Beutler M, Salman V, Schulz-Vogt H (2012). Unusual polyphosphate inclusions observed in a marine *Beggiatoa* strain. A van Leeuw J Microb.

[59] Berg JS, Schwedt A, Kreutzmann A-C, Kuypers MMM, Milucka J (2014). Polysulfides as intermediates in the oxidation of sulfide to sulfate by *Beggiatoa* spp. Appl Environ Microbiol.

[60] Høgslund S, Revsbech NP, Cedhagen T, Nielsen LP, Gallardo VA (2008). Denitrification, nitrate turnover, and aerobic respiration by benthic foraminiferans in the oxygen minimum zone off Chile. J Exp Mar Biol Ecol.

[61] Vairavamurthy A, Mopper K (1987). Geochemical formation of organosulphur compounds (thiols) by addition of H_2_S to sedimentary organic matter. Nature.

[62] Brassell SC, Lewis CA, de Leeuw JW, de Lange F, Sinninghe Damsté JS (1986). Isoprenoid thiophenes: novel products of sediment diagenesis?. Nature.

[63] de Graaf W, Sinningh Damsté JS, de Leeuw JW (1992). Laboratory simulation of natural sulfurization: I. formation of monomeric and oligomeric isoprenoid polysulfides by low-temperature reactions of inorganic polysulfides with phytol and phytadienes. Geochim Cosmochim Ac.

[64] Hofmann IC, Hutchison J, Robson JN, Chicarelli MI, Maxwell JR (1992). Evidence for sulphide links in a crude oil asphaltene and kerogens from reductive cleavage by lithium in ethylamine. Org Geochem.

[65] Kohnen MEL, Sinninghe Damsté JS, Rijpstra WIC and de Leeuw JW: Alkylthiophenes as sensitive indicators of palaeoenvironmental changes. In Geochemistry of Sulfur in Fossil Fuels, Edited by Orr WL, White, CM. American Chemical Society: Washington (DC); 1990:25.

[66] Sinninghe Damsté JS, van Duin ACT, Hollander D, Kohnen MEL, de Leeuw JW (1995). Early diagenesis of bacteriohopanepolyol derivatives: formation of fossil homohopanoids. Geochim Cosmochim Ac.

[67] Sinninghe Damsté JS, Kohnen MEL, Horsfield B (1998). Origin of low-molecular-weight alkylthiophenes in pyrolysates of sulphur-rich kerogens as revealed by micro-scale sealed vessel pyrolysis. Org Geochem.

[68] Ruttenberg KC (1992). Development of a sequential extraction method for different forms of phosphorus in marine sediments. Limnol Oceanogr.

[69] Soudry D, Champetier Y (1983). Microbial processes in the Negev phosphorites (southern Israel). Sedimentology.

[70] Jørgensen BB, Gallardo VA (1999). *Thioploca* spp.: filamentous sulfur bacteria with nitrate vacuoles. FEMS Microbiol Ecol.

[71] Soudry D, Glenn CR, Nathan Y, Segal I, Vonderhaar D (2006). Evolution of Tethyan phosphogenesis along the northern edges of the Arabian-African shield during the Cretaceous-Eocene as deduced from temporal variations of Ca and Nd isotopes and rates of P accumulation. Earth-Sci Rev.

[72] Niggemann J, Schubert CJ (2006). Fatty acid biogeochemistry of sediments from the Chilean coastal upwelling region: sources and diagenetic changes. Org Geochem.

[73] McCaffrey MA, Farrington JW, Repeta DJ (1989). Geochemical implications of the lipid composition of *Thioploca* spp. from the Peru upwelling region—15°S. Org Geochem.

[74] Gardosh M, Druckman Y, Buchbinder B and Rybakov M, The Levant basin offshore Israel: stratigraphy, structure, tectonic evolution and implications for hydrocarbon exploration, Geological Survey of Israel, 2008

[75] Almogi-Labin A, Ashckenazi-Polivoda S, Edelman-Furstenberg Y, Benjamini C, Altenbach AV, Bernhard JM, Seckbach J (2012). Anoxia-dysoxia at the sediment-water interface of the southern Tethys in the Late Cretaceous: Mishash Formation, southern Israel. Anoxia: Evidence for Eukaryote Survival and Paleontological Strategies, Cellular Origin, Life in Extreme Habitats and Astrobiology.

